# Unraveling the Diversity of Co-Colonization by CPE

**DOI:** 10.3390/microorganisms10071292

**Published:** 2022-06-25

**Authors:** Gabrielle Levi, Mor Lurie-Weinberger, Alona Keren-Paz, Antoine O. Andremont, David Schwartz, Yehuda Carmeli

**Affiliations:** 1 National Institute for Antibiotic Resistance and Infection Control, Ministry of Health, Tel Aviv 6423906, Israel; gabriellelev@cmft.gov.il (G.L.); morlw@tlvmc.gov.il (M.L.-W.); alonakp@tlvmc.gov.il (A.K.-P.); davidsc@tlvmc.gov.il (D.S.); 2 Microbiology Department, Université Paris Diderot, Sorbonne Paris Cité, 75018 Paris, France; antoine@andremont.fr; 3 Sackler Faculty of Medicine, Tel Aviv University, Tel Aviv 6997801, Israel

**Keywords:** co-colonization, CPE, CRE, HGT

## Abstract

Antibiotic-resistant bacteria, and more specifically, carbapenem-producing Enterobacterales (CPE) strains, are increasing worldwide. Despite their growing prevalence, in most high-income countries, the detection of CPE is still considered a low-frequency event. Sporadically, patients co-colonized with distinct CPE strains and/or different carbapenemase enzymes are detected. In this paper, we present three cases that illustrate the underlying mechanisms of co-colonization, focusing on horizontal gene transfer (HGT) and patient-to-patient transmission. We also demonstrate the diversity of CPE species and discuss the potential consequences of co-colonization.

## 1. Introduction

The emergence and spread of bacteria with multi-drug resistance (MDR) is a major public health concern worldwide, declared by the World Health Organization (WHO) as one of the top 10 global public health threats [1]. In 2019, more than 1.2 million deaths were directly attributable to bacterial MDR, with the overall death toll estimated at 4.95 million deaths [2]. Of the resistant bacteria listed as “critical” [3], carbapenem-resistant Enterobacteriaceae (CRE) are considered an urgent threat [4].

While some bacteria are naturally resistant to carbapenems, the rapid spread of CRE across the globe is mainly driven by acquired resistance, with susceptible bacteria gaining resistance by acquiring genes that encode for a variety of carbapenem-hydrolyzing enzymes [5], thus becoming carbapenem-producing Enterobacterales (CPE). This horizontal gene transfer (HGT) is mediated by various genetic elements, including plasmids, transposons [6], and integrons [7,8], and can occur between clones of a single species or between different species [9,10] within the human body. The gastrointestinal tract of hospitalized patients, with its diverse microbiota, is an ideal setting for HGT [11,12] promoting the spread of carbapenem resistance. Patients treated with carbapenems are particularly prone to becoming colonized by CRE, even if exposure is short [13,14]. Mobile genetic elements carrying resistance genes have been implicated in the patient-to-patient spread of resistant isolates and have been identified as the cause of many outbreaks [15,16]. Patient mobility has also been highlighted as a risk factor for CRE acquisition and spread [17], and the transfer of patients to different countries has been shown to introduce novel CRE variants into healthcare systems [18,19].

Typically, a single strain of CPE, carrying a single carbapenemase gene, is identified in a colonized patient. Occasionally, the co-carriage of two carbapenamases in the same strain is detected. However, as there is potential to acquire multiple CPEs exogenously (patient to patient), and for HGT to then occur across different species within the gastrointestinal tract, patients may have a substantial risk of becoming colonized with more than one CRE species. Whilst this phenomenon is now beginning to be investigated [20,21,22], these types of cases remain under-reported in the literature. Here, we describe in detail several such co-colonization cases with *Klebsiella pneumoniae* carbapenemase (KPC) and New Delhi metallo-beta-lactamase (NDM), focusing on the variety of the underlying genetic, microbiological, and epidemiological mechanisms.

## 2. Epidemiological Background from the National Center for Infection Control, Israel

Over the last 3 years, 95.6% of the total cases of CPE carriers reported to the National Registry of the National Centre for Infection Control (NCIC), Israel, had a single carbapenemase gene, while 4.4% of the CPE carriers had more than one type of carbapenemase. In 62% of those cases, the two carbapenemases were present in the same isolate, while in the remaining 38%, the different carbapenemases were present in different bacterial species.

## 3. Case Studies

**Figure 1:**
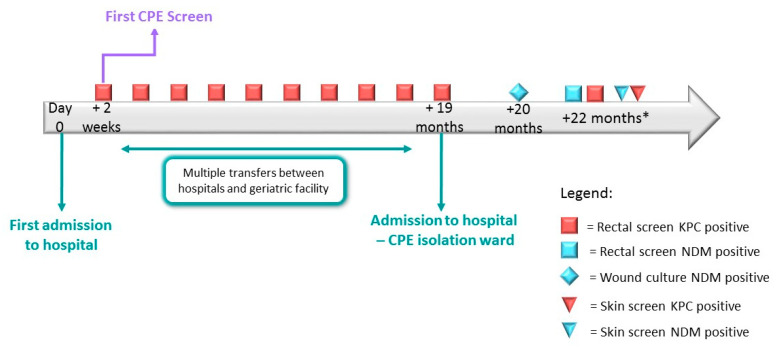
Epidemiological timeline of Case Study 1: patient’s previous known carbapenemase-producing Enterobacteriales (CPE) acquisitions and screening history, plus CPE results from point prevalence study in August 2021. * CPE detection performed by NCIC laboratory. Abbreviations: *Klebsiella pneumoniae* carbapenemase (KPC), New Delhi metallo-beta-lactamase (NDM).

In Case 1, a sixty-seven-year-old male was admitted to a hospital. In accordance with screening protocols, he was not screened on admission for CPE carriage as he had no risk factors. Two weeks after admission, routine screening revealed him to be CPE-positive, carrying *Klebsiella pneumoniae* carbapenemase (KPC)-producing *Enterobacter cloacae*. Subsequently, the patient had multiple hospitalizations and was transferred between different geriatric centers, and repeated CPE screening samples returned results positive for KPC-producing *Enterobacter cloacae* until the date of his next hospitalization, nearly two years later. The patient was hospitalized in a CPE cohort ward. The following month, a second CPE acquisition was identified—the patient’s wound culture was positive for New Delhi metallo-beta-lactamase (NDM)-producing *K. pneumoniae.* Two months later, whilst hospitalized in a geriatric medical center, he was screened for the presence of MDR bacteria, as part of a point prevalence study carried out by the NCIC. He was found to be positive for both KPC- and NDM-producing Enterobacterales. The rectal sample was found to contain NDM-producing *K. pneumoniae* and KPC-producing *K. pneumoniae*, whilst the skin sample contained NDM-producing *E. coli* and KPC-producing *K. pneumoniae* (Figure 1 and Table 1). Further WGS conducted by the NCIC laboratory confirmed that the patient was infected with two distinct *K. pneumoniae* isolates, carrying two different carbapenemase genes (Table 1).

**Figure 2:**
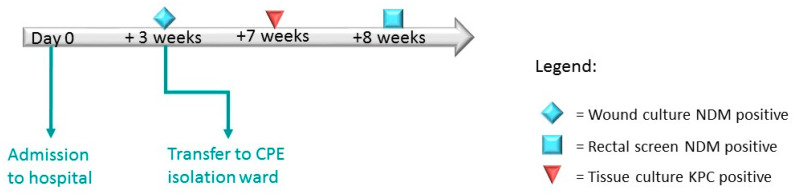
Epidemiological timeline of Case Study 2: patient’s CPE acquisition and screening results from period of hospitalization. All CPE detection performed by NCIC laboratory.

In Case 2, a 48-year-old man was admitted to an orthopedic department. Due to having no risk factors, this patient was not screened for CPE upon admission. Three weeks after admission, his surgical wound culture was identified as positive for NDM-producing *K. oxytoca*, and he was subsequently transferred to a CPE cohort ward. One month after his transfer, a tissue sample culture was identified as positive for KPC-producing *E. cloacae*. Concurrently, a rectal screening sample returned a positive result for NDM-producing *E. cloacae* (Figure 2 and Table 2). WGS analysis proved that the two *E. cloacae* isolates were distinct from one another (Table 2). Further analysis revealed that the *K. oxytoca* and the secondary *E. cloacae* carried the same variant of *bla*_NDM_. Strikingly, plasmid content analysis detected the presence of an IncX3 plasmid within both isolates (Table 2).

**Figure 3:**
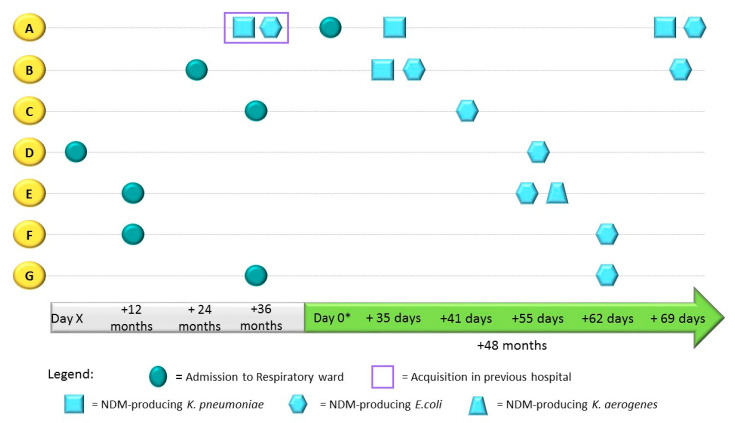
Epidemiological timeline of Case Study 3: patients A–G’s admission to hospital ward, CPE acquisition, and screening results spanning 5-year period. * From day 0 (start of outbreak), all patients were screened for CPE on a weekly basis, until receipt of a positive sample.

In Case 3, an outbreak of NDM-producing CPE in a ward of a post-acute care facility was detected. According to surveillance data reported to the NCIC, this was the first documentation of CPE transmission on this ward in 10 years. When patient A was admitted to the ward, he was known to have been previously colonized by NDM-producing *E. coli* and *K. pneumoniae*. Despite his CPE status, he was housed in a room with patient B. Shortly after patient A’s admission to the ward, patient B, who had been hospitalized for two years previous, was tested for CPE during routine screening and was found to be positive (Figure 3). Nursing staff at the NCIC were alerted, and point prevalence screening was then carried out weekly in the department in order to detect colonization and aid in the epidemiological investigation. Five additional patients (C–G) on the respiratory ward, some of whom shared a room with patient B, were subsequently found to be positive. For all these patients, this was the first positive result for CPE after one to four years of hospitalization (Figure 3). All positive screening samples were analyzed at the NCIC Reference Laboratory. Patient A’s initial rectal screen on admission was found to be positive for NDM-producing *K. pneumoniae*. Screening conducted one month later detected both NDM-producing *E. coli* and *K. pneumoniae.* Patient B’s rectal screen sample was found to be positive for NDM-producing *E. coli* and *K. pneumoniae.* Patients C–G were all found to be positive for NDM-producing *E. coli,* and patient E’s rectal screen showed NDM-producing *K. aerogenes* (Table 3, Figure 3). WGS analysis of the *E. coli* and *K. pneumoniae* from patient B showed that the two strains carried different *bla*_NDM_ variants (*bla*_NDM-7_ and *bla*_NDM-5_, respectfully). In addition, the *E. coli* strain contained an IncX3 plasmid. Further Sanger sequencing of the NDM-producing *E. coli* and *K. aerogenes* from patients C–G detected the presence of the *bla*_NDM7_ variant (Table 3) and showed that they had an IncX3 plasmid sharing a high degree of sequence identity with a previously reported plasmid, pC158-NDM7-IncX3 (Gen Bank accession: MN175471.1)

## 4. Methods

Case Study 1: Point prevalence study. Rectal and skin samples were used to inoculate liquid brain heart infusion (BHI) broth. After overnight incubation at 37 °C, the broth was sub-cultured on selective agar media for CPE (CHROMagar mSuperCARBA, Hy Laboratories Ltd., Rehovot, Israel). Both the BHI broth and suspect CPE colonies (when present) were tested for the presence of carbapenemase genes (including *bla*_KPC_, *bla*_NDM_, *bla*_IMI_, *bla*_VIM,_ and *bla*_OXA-48_-like) by multiplex polymerase chain reaction (PCR) based on Israeli National Policy for CPE Screening [23,24,25,26]. The presence of a carbapenemase was confirmed using a commercial ‘β-Carba Test’ kit (Biorad, Marnes-la-Coquette, France). Species identification and antibiotic susceptibility testing was performed using VITEK™ 2 (Biomerieux SA, Marcy I’Etoile, France). Whole genome sequencing (WGS) was performed on the NDM- and KPC-producing *K. pneumoniae.*

Case Studies 2 and 3: Upon receipt at the Reference Laboratory for Antibiotic Resistance (National Institute for Antibiotic Resistance, Ministry of Health), isolates were sub-cultured on CPE-selective media, and suspect CPE colonies were tested as outlined for Case Study 1. In Case 2, an additional immunochromatographic assay was performed, which was able to detect and differentiate between KPC, OXA-48-like, VIM, IMP, and NDM carbapenemases (NG-Test CARBA 5, NG Biotech, Guipry-Messac, France).

Whole genome sequencing (WGS): Sequencing was performed by the Sequencing Core at RUSH University, Chicago, IL, USA. Libraries were prepared using the Nextera XT (Illumina Inc., San Diego, CA, USA) kit, followed by sequencing on the Illumina NovaSeq (Illumina Inc.) using the high-output 2 × 150 bp kit (Illumina Inc.). De novo assembly was achieved using CLC Genomics Workbench protocol with default parameters. Strain MLST typing was determined with MLST software (https://github.com/tseemann/mlst (accessed on 5 May 2022)). The capsular polysaccharide (KL) and lipooligosaccharide outer core (OCL) synthesis was determined using Kaptive [27]. Antibiotic resistance genes were tested using the Center of Genomic Epidemiology ResFinder (https://cge.cbs.dtu.dk/services/ResFinder/ (accessed on 5 May 2022)). Plasmids were detected using PlasmidFinder (https://cge.food.dtu.dk/services/PlasmidFinder/ (accessed on 5 May 2022)). Sanger sequencing of *bla*_NDM_ PCR products was performed by Hy laboratories, Israel.

Institutional review board approval was not required for this study.

## 5. Discussion

The most important finding of this study demonstrates that co-colonization with different CPEs may occasionally occur and is often detected by screening patients in cohort wards or patients who have a high risk of CPE carriage. It involves a high diversity of species, thus increasing the potential for further transmission and spread of the carbapenemase genes into bacterial species previously susceptible to carbapenems. This is because one patient can acquire multiple CPEs through patient-to-patient transmission, or a patient can acquire a CPE and subsequently experience HGT across different species within the gastrointestinal tract.

In Cases 1 and 2, the two patients had no previously known or documented risk factors for CPE upon hospitalization, but were likely colonized (Case 1) or infected (Case 2) with CPE during their stay in the hospital. The combination of the documented screening results in Case 1 and the positive CPE results from both patients obtained by the NCIC Reference Laboratory allows us to conclude with high certainty that both secondary CPE acquisitions of a different carbapenemase gene occurred on the CPE isolation ward. This demonstrates that the inappropriate cohorting of patients with different carbapenemases is a risk factor for co-colonization with CPE and can lead to the acquisition of additional resistance mechanisms by previously resistant or susceptible bacteria.

Another example of the results of failure to isolate a CPE carrier was reported in Case Study 3. In this case, an outbreak was caused by a clonally related *bla*_NDM7_-producing *E. coli* strain, transmitted between six patients and leading to their colonization. Most noteworthy in Case Study 3, however, is the clear evidence of HGT, which occurred in patient E. The rapid global spread of *bla_NDM_* in different bacterial species is extensively reported in the literature, because this gene is carried on a broad host range of conjugative plasmids [28,29]. Here, we discuss two cases of HGT occurring between distinct bacterial species that were very likely mediated by a plasmid: (1) *bla*_NDM-7_ was transferred between IncX3-carrying *E. coli* and *K. aerogenes* (Case 3) and (2) *bla*_NDM-1_ was transferred between *K. oxytoca* and *E. cloacae* (Case 2), both carrying IncX3. Despite the lack of detailed data on all CPE-positive isolates in Case Study 1, it is possible that HGT occurred twice in this patient: (1) *bla*_KPC-3_ was transferred between *E. cloacae* and *K. pneumoniae* ST512, and (2) *bla*_NDM-1_ was transferred between *K. pneumoniae* ST417 and *E. coli*. The repetition of this event in our results suggests that cross-species transmission occurs more frequently than is currently believed, especially during co-colonization events [20].

One reason for the underestimation of the frequency of co-colonization events is the in-depth WGS analysis needed for its detection. The WGS analysis performed by NCIC Reference Laboratory identified two distinct NDM variants (*bla*_NDM-7_ and *bla*_NDM-5_) within three different bacterial species in Case 3. Additionally, the WGS analysis of isolates from Case 1 and 2 identified two distinct *K. pneumoniae* and two different *E. cloacae* strains, belonging to different ST types. In each case, the different strains carried different carbapenemase genes. Without sequencing to determine the exact ST type and the specific allele within each species, the complex picture of co-colonization would have remained unclear.

The diversity of co-colonization described here highlights the importance of sub-classifying patients according to the carbapenemase genes carried by their CPE strains, rather than just according to the detection of CPE. Previous studies have suggested that multiple colonization with CPE may increase the risk of further infection with these organisms [21]. This could drastically lower patient outcomes, increasing morbidity and mortality due to complicated infections with very limited treatment options. The variety of species, carrying different carbapenemase genes, present in the same crowded niche (the GI tract), may carry a substantial risk of simultaneous colonization with more than one CPE species. The increasing prevalence of CPE worldwide [30,31,32] further increases this risk.

The inappropriate isolation and cohorting of patients with different resistance mechanisms increases the likelihood of co-colonization. Therefore, to improve patient outcomes, patients should be separated whenever feasible. Not detecting the presence of CPE allows transmission to occur, increasing the risk of co-colonization. The screening of patients with CPE or those who are at risk of CPE is essential for detecting changes in colonization status and allows fast action to prevent transmission or acquisition.

There are several limitations to this study. Not all isolates were available for WGS and plasmid extraction, and therefore the genetic analysis was not complete. In addition, the identification of all potential CPE species from screening swabs is subject to microbiological and technical limitations, and therefore the CPE detection results may have been underestimated.

## 6. Conclusions

We demonstrate here that there is no single CPE status: multiple different CPE strains may be present in a single CPE-positive patient. The diversity of resistance genes and the presence of natural HGT mechanisms increases the potential of the spread of multiple carbapenemases between different currently susceptible bacterial species, allowing the possible combinations of CPE to become infinite. It is therefore of vital importance to continue global efforts to control the spread of these MDR organisms through appropriate cohorting, increased screening, and performing real-time in-depth genetic analysis in clinical settings.

## Figures and Tables

**Table 1:** Summary of results for patient in Case Study 1—CPE positive bacteria, isolated in the point prevalence study performed by the National Centre for Infection Control (NCIC).

Sample Type	Carbapenemase Detected	Bacterial Identification	‘β-Carba Test’	ST Type	KL Type	O Type
Rectal swab	*bla*_NDM-1_	*K. pneumoniae*	Positive	417	64	O1/O2v1
	*bla*_KPC-3_	*K. pneumoniae*	Positive	512	107	O1/O2v2
Skin culture	NDM	*E. coli*	Positive	Unknown	Unknown	Unknown
	*bla*_KPC-3_	*K. pneumoniae*	Positive	512	107	O1/O2v2

**Table 2:** Summary of results of CPE-positive bacteria isolated during routine CPE screening in Case Study 2.

Sample Type	Carbapenemase Detected	Bacterial Identification	‘β-Carba Test’	NG-Test CARBA 5	ST Type (Pasteur)	Presence of IncX3 Plasmid
Surgical wound	*bla*_NDM-1_	*K. oxytoca*	Positive	NDM	202	+
Tissue culture	*bla*_KPC-2_	*E. cloacae*	Positive	KPC	88	−
Rectal swab	*bla*_NDM-1_	*E. cloacae*	Positive	NDM	145	+

**Table 3:** Summary of results of CPE-positive bacteria isolated during CPE screening in Case Study 3.

Patient	Date of Screen	Carbapenemase Detected	Bacterial Identification	‘β-Carba Test’	NDM Variant	Presence of IncX3 Plasmid
A	4 April 2022	NDM	*K. pneumoniae*	Positive	*bla*_NDM-5_	−
	8 May 2022	NDM	*E. coli*	Positive	_*bla*NDM-7_	+
		NDM	*K. pneumoniae*	Positive	*bla*_NDM-5_	-
B	4 April 2022	NDM	*E. coli*	Positive	*bla*_NDM-7_	+
		NDM	*K. pneumoniae*	Positive	*bla*_NDM-5_	-
	8 May 2022	NDM	*E. coli*	Positive	*bla*_NDM-7_	+
C	10 April 2022	NDM	*E. coli*	Positive	*bla*_NDM-7_	+
D	24 April 2022	NDM	*E. coli*	Positive	*bla*_NDM-7_	+
E	24 April 2022	NDM	*E. coli*	Positive	*bla*_NDM-7_	+
		NDM	*K. aerogenes*	Positive	*bla*_NDM-7_	+
F	1 May 2022	NDM	*E. coli*	Positive	*bla*_NDM-7_	+
G	1 May 2022	NDM	*E. coli*	Positive	*bla*_NDM-7_	+

## Data Availability

All sequence data were deposited at the NCBI repository under BioProject PRJNA843363 (see Supplementary Materials for sequence data information).

## Supplementary Materials

The following supporting information can be downloaded at: https://www.mdpi.com/article/10.3390/microorganisms10071292/s1. Table S1. sequence data information.
